# Predicting Discharge to Institutional Long‐Term Care After Stroke: A Systematic Review and Metaanalysis

**DOI:** 10.1111/jgs.15101

**Published:** 2017-10-09

**Authors:** Jennifer K. Burton, Eilidh E. C. Ferguson, Amanda J. Barugh, Katherine E. Walesby, Alasdair M. J. MacLullich, Susan D. Shenkin, Terry J. Quinn

**Affiliations:** ^1^ Alzheimer Scotland Dementia Research Centre University of Edinburgh Edinburgh United Kingdom; ^2^ Centre for Cognitive Ageing and Cognitive Epidemiology University of Edinburgh Edinburgh United Kingdom; ^3^ Department of Medicine for the Elderly Royal Infirmary of Edinburgh National Health Service Lothian Edinburgh United Kingdom; ^4^ Geriatric Medicine University of Edinburgh Edinburgh United Kingdom; ^5^ Institute of Cardiovascular and Medical Science University of Glasgow Glasgow United Kingdom

**Keywords:** stroke, long‐term care, hospitalization, predictors

## Abstract

**Background/Objectives:**

Stroke is a leading cause of disability worldwide, and a significant proportion of stroke survivors require long‐term institutional care. Understanding who cannot be discharged home is important for health and social care planning. Our aim was to establish predictive factors for discharge to institutional care after hospitalization for stroke.

**Design:**

We registered and conducted a systematic review and meta‐analysis (PROSPERO: CRD42015023497) of observational studies. We searched MEDLINE, EMBASE, and CINAHL Plus to February 2017. Quantitative synthesis was performed where data allowed.

**Setting:**

Acute and rehabilitation hospitals.

**Participants:**

Adults hospitalized for stroke who were newly admitted directly to long‐term institutional care at the time of hospital discharge.

**Measurements:**

Factors associated with new institutionalization.

**Results:**

From 10,420 records, we included 18 studies (n = 32,139 participants). The studies were heterogeneous and conducted in Europe, North America, and East Asia. Eight studies were at high risk of selection bias. The proportion of those surviving to discharge who were newly discharged to long‐term care varied from 7% to 39% (median 17%, interquartile range 12%), and the model of care received in the long‐term care setting was not defined. Older age and greater stroke severity had a consistently positive association with the need for long‐term care admission. Individuals who had a severe stroke were 26 times as likely to be admitted to long‐term care than those who had a minor stroke. Individuals aged 65 and older had a risk of stroke that was three times as great as that of younger individuals. Potentially modifiable factors were rarely examined.

**Conclusion:**

Age and stroke severity are important predictors of institutional long‐term care admission directly from the hospital after an acute stroke. Potentially modifiable factors should be the target of future research. Stroke outcome studies should report discharge destination, defining the model of care provided in the long‐term care setting.

Stroke unit care reduces the likelihood of death and the need for long‐term care,^1^ but admission to long‐term care after stroke is common, with approximately 26% of stroke survivors living in long‐term care after 6 months in the United States^2^ and 19% of stroke survivors requiring long‐term care admission within 5 years in the United Kingdom.^3^ Stroke survivors living in long‐term care often have significant persistent functional and cognitive impairments.^4^ Admission to long‐term care is costly for individuals and society.^5^, ^6^ Understanding the determinants of long‐term care admission could potentially identify targets for intervention to reduce institutional care.

Predictors of admission to institutional long‐term care in the older adult population and those with dementia include low self‐rated health, caregiver burden, dependence in activities of daily living, cognitive impairment, and polypharmacy,^7^, ^8^ but stroke is an unpredictable, sudden condition, and factors that predict this outcome for stroke survivors may be different. Many studies use composite measures of “poor” stroke outcome, combining institutional care admission with disability or death, but clear prognostic predictors have not been established from the available data.^9^ Individuals and their families may place different values on these outcomes, and understanding predictors of institutional care as a single outcome is important for planning rehabilitation and for clear communication. Understanding which stroke survivors require long‐term care placement directly after hospital admission for a stroke is particularly important for health and social service planning.

Our aim was to perform a systematic review of predictive factors for new admission to long‐term care directly after hospitalization for stroke.

## Methods

This review was reported in accordance with the Preferred Reporting of Items in Systematic Reviews and Meta‐Analyses guidance.^10^ The protocol was registered on August 20, 2015: (CRD42015023497; http://www.crd.york.ac.uk/PROSPERO/display_record.asp?ID=CRD42015023497).

### Eligibility Criteria

Studies were eligible if they were observational, included participants of any age hospitalized for stroke, and had data on factors associated with long‐term care admission directly from this admission. We were interested in the natural distribution of characteristics in the population, so intervention studies were excluded. The search was designed to identify unselected admissions, and stroke‐specific articles were then selected.

The exposure of interest was any factor that any of the individual studies examined as a possible predictor. The outcome of interest was admission directly to a long‐term care setting as the new place of residence at hospital discharge, excluding admissions to long‐term care after an interval or instances in which the outcome was evaluated at a fixed follow‐up point after discharge because changes in residence could have occurred in the interim.

No restrictions were placed on date or language of publication. If abstracts were identified, we searched for subsequent full‐text publications and contacted the authors if full texts were not available.

### Information Sources

We searched MEDLINE databases (Ovid); EMBASE (Ovid), and CINAHL Plus (EBSCOhost) from inception to September 2015. An update search was performed on February 13, 2017.

The search was developed with the expertise of an information specialist from the University of Edinburgh Library. The full strategy is in Supplementary Text S1. This was supplemented by a review of reference lists from systematic reviews and a forward citation search.

### Study Selection and Data Collection

Three authors JKB, AJB, KEW independently screened all titles and abstracts using Covidence software (Melbourne, Australia).^11^ Independent pairs of reviewers evaluated full texts and resolved conflicts by discussion. A data extraction form was developed and piloted to improve usability. A single author JKB extracted data, with co‐authors double‐extracting on a random sample of 33%.

Data were extracted on sample size, country, study design, data collection period, setting, age, sex, proportion of those surviving to discharge who were discharged to new long‐term care, dementia diagnosis and, reporting of other comorbidities. Additional information was extracted to complete the risk‐of‐bias assessment. All reported predictors were recorded.

### Risk‐of‐Bias Assessment

Risk of bias was assessed based on the Risk of Bias Assessment Tool for Non‐Randomized Studies.^12^ (Supplementary Text S2).

### Summary Measures

Studies were included if they reported quantitative data with statistical tests of association, including reporting of risk ratios (RRs), odds ratios (ORs), correlations, and differences in proportion between two groups with comparative significance testing.

### Synthesis of Results

Quantitative analysis was performed using Comprehensive Meta‐Analysis software.^13^ Where data were reported on the same predictor from three or more studies, summary estimates were calculated. Random‐effects models were used to calculate pooled ORs and 95% confidence intervals (CIs). These data were evaluated using the Grading of Recommendations Assessment, Development and Evaluation approach to describe the quality of the evidence.^14^ This rates evidence as being of high, moderate, low, or very low quality, starting at high and downgrading for recognized parameters that can reduce the quality of a body of evidence, including risk of bias, heterogeneity, and imprecision.^14^ Because we sought only observational studies, we did not downgrade the quality of the evidence for this factor. We categorized stroke severity using the National Institute for Health Stroke Scale (NIHSS) as minor (<6), moderate (6–15), moderate to severe (16–20), and severe (≥21).^15^ If data were not reported exactly within a category, we used the category that best aligned with the data presented in the original article.

Planned subgroup analyses included type of care provided (residential or nursing), country of origin, age, and presence of dementia or delirium.

## Results

**Figure 1:**
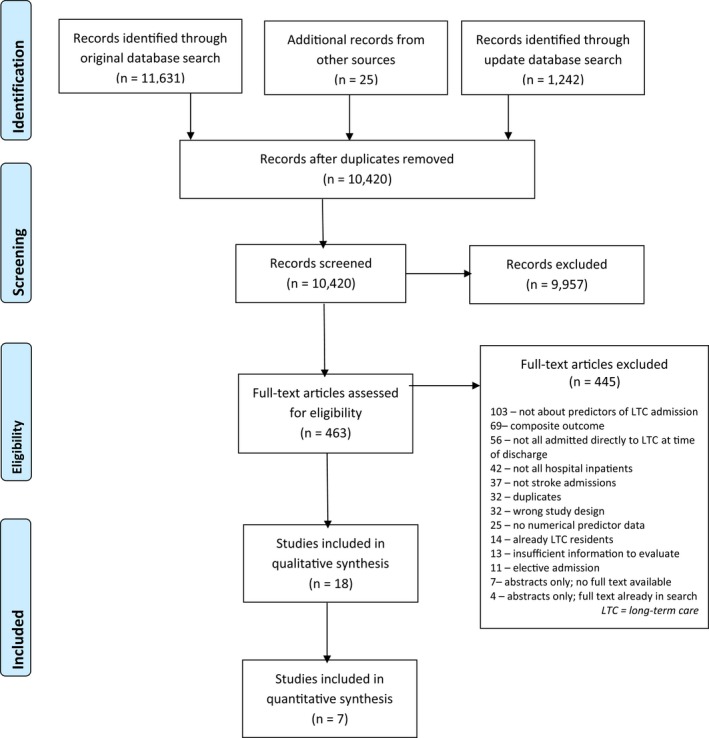
Preferred Reporting of Items in Systematic Reviews and Meta‐Analyses flow diagram. [Color figure can be viewed at wileyonlinelibrary.com]

We identified 10,420 records after initial de‐duplication, read 463 full texts, and included 18 cohort studies: 11 prospective and seven retrospective (Figure 1),^16^, ^17^, ^18^, ^19^, ^20^, ^21^, ^22^, ^23^, ^24^, ^25^, ^26^, ^27^, ^28^, ^29^, ^30^, ^31^, ^32^, ^33^ including one study that we translated from Spanish.^27^

The total study population included 32,139 individuals admitted to the hospital with acute stroke, sample sizes varied from 82 to 21,575 (median 412, interquartile range (IQR) 931) (Table 1, Supplementary Table S3). Ten studies were conducted in Europe, six in North America, and two in East Asia. Median study duration was 18 months (range 2–77 months; not reported in one study).

**Table 1:** Characteristics of Included Studies

Author, Year	N	Country	Study Design	Design	Duration, Months	Inpatient Death	Discharged to Care Home
						% (n/N)	
Béjot, 16	1,069	France	Prospective	Population registry	48	15 (156/1069)	15 (140/913)
Brosseau, 17	152	Canada	Prospective	Cohort	15	NR	23 (35/152)
Ifejika, 18	346	United States	Retrospective	Registry	34	NR	14 (47/346)
Kammersgaard, 19	1,156	Denmark	Prospective	Cohort	NR	20 (236/1,156)	26 (236/920)
Koyama, 20	163	Japan	Prospective	Cohort	18	NR	25 (40/163)
Kwan, 21	439	United Kingdom	Prospective	Cohort	10	12 (54/439)	39 (150/381)
Lai, 22	662	United States	Prospective	Cohort	24	3 (22/692)	19 (128/660)
McManus, 23	82	United Kingdom	Prospective	Cohort	7	10 (8/82)	14 (10/74)
Murie‐Fernández, 24	536	Canada	Retrospective	Cohort	44	NR	28^a^
Pérez, 32	384	Spain	Prospective	Cohort	12	NR	25 (89/353)
Pinedo, 25	241	Spain	Prospective	Cohort	8	NR	19
Portelli, 26	2,778	United Kingdom	Retrospective	Audit	2	29 (812/2,778)	14
Ramirez‐Moreno, 27	130	Spain	Retrospective	Hospital registry	26	NU 14 Other 17	NU 11 Other 30
Rundek, 28	573	United States	Prospective	Cohort	77	5 (31/604)	9 (54/573)
Schlegel, 29	94	United States	Retrospective	Cohort	4	10 (12/119)	12 (11/94)
Treger, 30	1,583	Israel	Prospective	National survey	2	8 (162/2,174)	7 (117/1,583)
Tseng, 33	21,575	Taiwan	Retrospective	Registry study	24	NR	7 (1,397/21,575)
Turco, 31	176	Italy	Retrospective	Cohort	47	11 (20/176)	27 (42/156)

a Composite of institution and assisted living facility, individual data not reported.

NR = not reported; NU = neurology unit.

The mean age of participants ranged from 58.9 to 88.9, and the majority of participants were female in most studies. Baseline stroke severity was reported in only 10 studies using the NIHSS, the Scandinavian Stroke Scale, and the Functional Independence Measure. Only five studies provided data on the race or ethnicity of included participants.^18^, ^22^, ^26^, ^28^, ^34^ None of the studies reported on the socioeconomic status of included participants.

One study compared the effect of receiving specialist neurology care with that of general ward care as a predictor of outcomes.^27^ Seven studies reported no exclusion criteria.

The proportion of inpatient deaths was reported in 11 studies and varied from 3% to 29% (median 11%, IQR 7.5). The proportion of those surviving to discharge who were newly discharged to long‐term care ranged from 7% to 39% (median 17%, IQR 12).

### Risk of Bias in Studies

Eight studies were considered to be at high risk of selection bias because of limited generalizability (Supplementary Figure S1), particularly through exclusion of those with prior stroke, those who were younger, and those who had other comorbidities, including cognitive impairment.^19^, ^21^, ^22^, ^24^, ^27^, ^28^, ^29^, ^31^ All studies were assessed as at unclear risk of reporting bias because of the unavailability of study protocols. Five studies were considered to be at low risk of bias for all other domains.^15^, ^16^, ^17^, ^18^, ^30^

### Quantitative Results

#### Studies Reporting Multivariate ORs and RRs of Predictors

**Figure 2:**
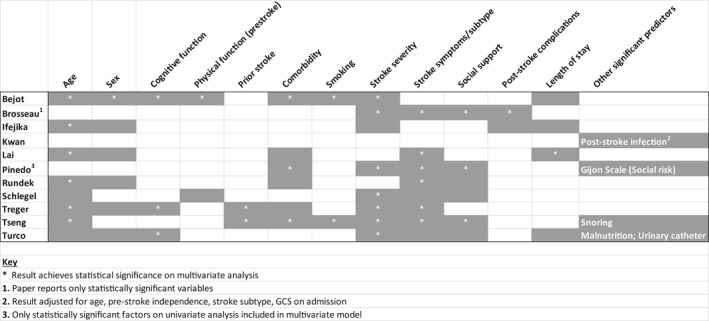
Multivariate predictors evaluated.

**Figure 3:**
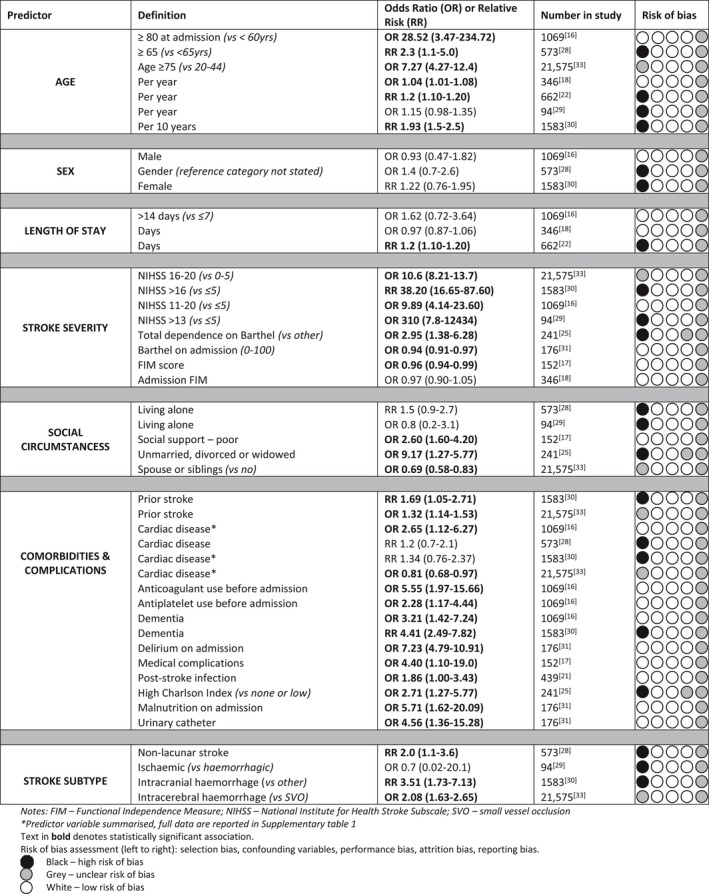
Summary of multivariate predictors of long‐term care admission from 11 studies.

Eleven studies presented multivariate models with ORs and RRs of predictors.^16^, ^17^, ^18^, ^21^, ^22^, ^25^, ^28^, ^29^, ^30^, ^31^, ^33^ Figure 2 summarizes the factors that the individual studies considered, indicating where statistically significant associations were identified. Age; sex; and stroke severity, symptoms, and subtype were the most commonly examined. Their effect sizes are reported in Figure 3 and Supplementary Table S2 None of the studies evaluated patient or family preferences, socioeconomic status, availability of social care, costs of care, insurance status, dysphagia, or continence. Two studies reported data only when statistically significant associations were identified.^17^, ^25^

#### Age

Older age was associated with greater likelihood of long‐term care admission. Illustrative synthesis of the available data comparing participants aged 65 and older (or nearest available) with those younger than 65 is OR = 3.88 (95% CI = 1.38–6.40; three studies; 23,217 participants; low‐quality evidence downgraded because risk of bias and imprecision) and the summary OR per year increase in age was 1.12 (95% CI = 1.00–1.24, three studies; n = 1,102 participants; moderate‐quality evidence downgraded because risk of bias).

#### Sex

There was no evidence of sex as a predictor of the need for long‐term care (n = 3,225).^16^, ^28^, ^30^

#### Length of Stay

Longer stay was associated with long‐term care admission in one study, not adjusted for stroke severity (n = 662),^22^ with no association seen in those studies which accounted for stroke severity (n=1,415).^16^, ^18^

#### Stroke Severity

Greater stroke severity, determined according to NIHSS score, was associated with admission to long‐term care. Participants with a NIHSS score of 6 to 10 (OR = 2.9, 95% CI = 2.4–3.6; three studies; 24,227 participants; moderate‐quality evidence downgraded because of risk of bias), 11 to 16 (OR = 9.3, 95% CI = 5.4–16.0; four studies; 24,321 participants; low‐quality evidence downgraded because of risk of bias and heterogeneity), and 21 or greater (OR = 26.6, 95% CI = 12.6–56.3; three studies; 24,227 participants; very‐low‐quality evidence downgraded because of imprecision, heterogeneity and risk of bias) were more likely to be admitted to long‐term care than those with a score of 0 to 5. The risk of admission to long‐term care was three times as great in participants with total functional dependence (Barthel Index score 0–20) (n = 241).^25^

#### Social Support

Being unmarried, divorced, or widowed or having “poor social support” was associated with greater likelihood of admission to long‐term care in two studies (n = 393),^17^, ^25^ with no evidence of an association between this outcome and living alone in two others (n = 667).^28^, ^29^ Having an employed caregiver was associated with greater likelihood of long‐term care admission, whereas having a spouse or siblings (not necessarily as a caregiver) was associated with lower risk in one large study (n = 21,575).^33^

#### Comorbidities

Previous stroke was associated with admission to long‐term care (n = 23,158).^30^, ^33^ People with comorbidities^26^, ^34^ (n = 21,816) or complications during their inpatient stay^17^, ^21^, ^31^ (n = 767) had higher rates of long‐term care admission.

#### Stroke Subtype

Hemorrhagic stroke was associated with admission to long‐term care (n = 23,158), accounting for age and stroke severity.^29^, ^32^

### Studies Reporting Other Data Analysis

A study that used logistic regression to determine parameter estimates with their standard errors found that age, FIM score, number of household members, and presence of a spouse were associated with admission to long‐term care (n = 163).^20^

Another study used univariate analysis, but did not report multivariate model results, instead discussing the extent of variation in outcome explained by age, length of stay and function (using BI). They identified that 54% and 22% of variation in outcome was due to total Glasgow Coma Scale and ability to walk unaided (n=2,778).^26^

Two studies report unadjusted analyses of a single predictor.^23^, ^27^ Delirium within 14 days of admission was associated with greater risk of long‐term care admission (OR = 14, 95% CI = 3.05–64.37) (n = 82).^23^ Treatment in a neurology ward rather than a general ward was associated with lower risk of long‐term care admission (*P* = .006) (n = 130).^27^

Another study identified a correlation of 0.308 (*P* < .01) between delay in starting neurorehabilitation and the need for admission to long‐term care at discharge (n = 536).^24^

One study found no statistically significant association between early infection and long‐term care admission (n = 1,156).^19^

Another study defined rehabilitation complexity, incorporating age, prestroke function, comorbidity, severity, cognition, and disability with the presence or absence of a caregiver.^32^ A greater proportion of participants with higher rehabilitation complexity and a caregiver experienced long‐term care admission than of those with lower complexity and a caregiver (*P* = .008), although this was no longer statistically significant when adjusted (*P* = .14) (n = 384).^32^

### Subgroup Analyses

Lack of useable data precluded our planned subgroup analyses describing the effects of age, country of origin, and residential versus nursing care. None of the studies had a mean age of younger than 65 for the entire study population. In the small number of included countries, no differences were identified between countries in predictors of long‐term care. No studies considered differences in the level of care provided in the long‐term care institution.

### Dementia and Delirium

Two studies with a total population of 2,652 individuals found with multivariate analysis that the risk of long‐term care admission was three to four times as great in individuals with diagnosed dementia.^15^, ^29^ Delirium was also examined in two smaller studies that found evidence of a positive association, with estimates that the risk was from seven times as great (n = 176, multivariate analysis)^31^ to 14 times as great (n = 82, unadjusted analysis).^23^

## Discussion

In 18 studies of predictors of long‐term care admission directly after hospitalization for stroke, there was marked heterogeneity in study size, stroke mortality, rate of discharge to long‐term care, and predictors reported.

There was a consistently positive association between older age and long‐term care admission at discharge after stroke. Greater stroke severity was also associated with greater risk, although the quality of the evidence was low. The use of different measures of stroke severity and subtype and less‐formal descriptions of stroke symptoms or deficits limited quantitative synthesis.

The role of dementia and delirium were explored in only four studies, all of which found greater risk of discharge to long‐term care. Cognitive impairment has been identified as a predictor of institutionalization over longitudinal follow‐up after stroke, although previous research has incorporated assessment of severity of cognitive impairment^35^ that the studies included in this review did not report. Delirium is recognized as a common complication after stroke and is associated with institutionalization;^36^ it is possible to prevent up to one‐third of cases in hospitalized individuals,^37^ although there is no specific evidence yet for delirium prevention interventions after stroke.

Social support was included in five studies, but these all used very different definitions. Longitudinal data has identified the availability of social support as an important predictor of the likelihood of requiring long‐term care,^38^ and this appears plausible. None of the studies considered the role of individual or family choice or the availability of social care.

Age and stroke severity are not modifiable risk factors; it is striking that potentially modifiable predictors were not widely examined. The level of disability (which may include, but is not limited to, stroke severity) after stroke has been described as a predictor of long‐term care admission.^2^, ^38^ The role of organized stroke unit care in reducing the need for institutional care has been confirmed in routine clinical practice.^39^ Prevention and treatment of complications, such as infections and pressure ulcers, as part of routine stroke care are associated with lower risk of death.^40^ Medical complications and poststroke infection were associated with long‐term care admission, and these may be important targets for preventative intervention.

Other factors pertaining to the costs of long‐term care such as insurance status or socioeconomic status and stroke specific‐factors such as dysphagia^41^ were not evaluated.

Variation in rates of long‐term care admission is also recognized in individuals who have not had a stroke.^42^ The rate of long‐term care admission from the hospital can be used for benchmarking stroke services across a region.^43^

### Strengths and Limitations

This review addresses a pragmatic need to identify individuals who require institutional long‐term care admission after hospitalization for stroke. It sought examples from observed practice and included studies based in acute and rehabilitation settings. The search strategy was inclusive, seeking initially to identify all studies evaluating predictors of institutionalization after hospitalization and identifying stroke as a relevant subgroup.

The review does not include studies that assessed for the outcome of interest at a fixed period of follow‐up, but future reviews could determine whether predictors of long‐term care admission differ after discharge. We sought studies in which long‐term care was the place of permanent residence, although the composition of care services provided and terminology in this area vary internationally^44^ so it is possible that some settings provided rehabilitation. Several studies used a composite outcome of death and institutionalization or institutionalization combined with other outcomes and were therefore excluded.

### Quality of Evidence

The main risk of bias was the selection of participants, particularly through exclusion of those with a prior stroke, who have a higher risk of institutionalization.^45^ The absence of published protocols for any of the studies, which is common in observational research, meant that reporting bias could not be assessed. Lack of standardization in reporting and classifying potential predictor variables and incomplete reporting whereby many papers on reported statistically significant associated limited metaanalysis. Estimates for age are presented as illustrative rather than definitive values because of the assumptions required to extrapolate from the underlying data. Results of the quantitative synthesis are imprecise, reflecting significant variation in the sample sizes of the included studies. This results in the quantitative data being downgraded to moderate to very low quality.

None of the included studies reported data from any low‐ or middle‐income countries, where the incidence of stroke is growing most rapidly,^45^ so the findings cannot be considered to have global generalizability.

### Implications for Practice

Older adults with greater severity of stroke are at higher risk of requiring long‐term care at the time of hospital discharge. This can help to inform prognosis and discussions with patients and their families. Many factors that are likely to influence this decision (e.g., patient and family views, support network, cognitive function, continence, progress with rehabilitation) were not included in published studies and need to be explored in more detail. Acute and rehabilitation services need to be staffed and resourced to address these needs. There needs to be a greater understanding around the extent to which this outcome can be modified. Rates of long‐term care admission directly from inpatient hospital settings may be a metric to compare stroke services, but interpreting this will require greater understanding of the variability in services provided in hospitals, in funding models, in long‐term care facilities, and in individuals admitted to them.

### Implications for Research

If reducing admission to long‐term care is important to patients, caregivers, and service providers, future research should explore potentially modifiable risk factors, such as delirium and infection, and efforts to reduce total disability. Randomized trials of interventions in stroke must separate the outcome of long‐term care admission from death and disability. What constitutes long‐term care in the local healthcare setting should be clearly described. Better use of the existing validated measures to describe stroke symptoms and outcomes would facilitate comparison between studies and allow pooling of data. Consideration of the role of social factors such as availability of care, costs, and social support would also help ensure a more‐comprehensive understanding of the determinants of institutionalization after stroke. Finally, if long‐term care admission is used as a stroke trial outcome, this review identifies that baseline characteristics need to be accounted for when evaluating the effects of an intervention.

## Supporting information


**Text S1:** Search Strategy
**Text S2:** Quality Assessment Criteria
**Table S1:** Additional included study population characteristics
**Table S2:** Complete multivariate predictors of long‐term care admission from 11 studies
**Figure S1:** Risk of Bias Summary Chart
