# Supplementary material for: Predicting Discharge to Institutional Long‐Term Care After Stroke: A Systematic Review and Metaanalysis
Source: J Am Geriatr Soc. 2017 Oct 9;66(1):161–9. doi: 10.1111/jgs.15101 (PMC5813141; doi:10.1111/jgs.15101)
Supplement: Supplementary file 1 — Text S1: Search Strategy Text S2: Quality Assessment Criteria Table S1: Additional included study population characteristics Table S2: Complete multivariate predictors of long‐term care admission from 11 studies Figure S1: Risk of Bias Summary Chart [file JGS-66-161-s001.docx]

**Supplementary materials**

**Supplementary Text S1: Search Strategy**

**Supplementary Text S2: Quality Assessment Criteria**

**Supplementary Table S1: Additional included study population characteristics**

**Supplementary Figure S1: Risk of Bias Summary Chart**

**Supplementary Table S2: Complete multivariate predictors of long-term care admission from 11 studies**

**Supplementary Text S1: Search Strategy**

*The final search for each database combined: Concepts 1, 2 & 3 with AND*

Ovid MEDLINE (R) In Process and Other Non-Indexed Citations and Ovid MEDLINE (R) 1946 to present

Search Concept 1: Care home

nursing home.mp.2. OR (care home or care home*).mp. OR Residential Facilities/ or residential.mp. OR institutionalisation.mp. OR institutionalization.mp. or exp Institutionalization/ OR "long term care".mp. or Long-Term Care/ OR exp Homes for the Aged/ OR ((aged or elderly) adj2 (care or facilit* or home*)).ti,ab.

Search Concept 2: Hospitalisation

Hospitalisation: hospital.mp. or Hospitals/ OR inpatient.mp. or Inpatients/ OR in-patient.mp. OR hospitalisation.mp. OR Hospitalization/ or hospitalization.mp. OR hospitalised.mp.

Search Concept 3: Risk/predictive factor

Risk/predictive factor: risk factor.mp. or exp Risk Factors/ OR cause.mp. OR causes.mp. OR predict.mp. OR predictor.mp.

Ovid EMBASE 1980 to 2015 Week 39

Search Concept 1: Care home

nursing home.mp. or nursing home/ OR care home.mp. OR care home*.mp. OR residential care/ or residential.mp. or residential home/ OR institutionalisation.mp. OR institutionalization.mp. or exp institutionalization/ OR long term care.mp. or long term care/ OR exp home for the aged/ OR ((aged or elderly) adj2 (care or facilit* or home*)).ti,ab.

Search Concept 2: Hospitalisation

hospital/ or hospital.mp. OR inpatient.mp. OR in-patient.mp. OR hospitalisation.mp. OR hospitalization/ or hospitalization.mp. OR hospitalised.mp.

Search Concept 3: Risk/predictive factor

risk factor.mp. or exp risk factor/ OR cause.mp. OR causes.mp. OR predict.mp. OR predictor.mp.

EBSCOhost CINAHL Plus

Search Concept 1: Care home

(MH “Nursing Homes”) OR “nursing home” OR “care home*” OR “care home” OR (MH “Residential Care”) OR “residential” OR (MH “Residential Facilities”) OR “institutionalisation” OR (MH “Institutionalization”) OR “institutionalization” OR (MH “Long Term Care”) OR “long term care”

Search Concept 2: Hospitalisation

“hospital” OR (MH “Hospitals”) OR “inpatient” OR (MH “Inpatients”) OR “in-patient” OR “hospitalisation” OR (MH “Hospitalization”) OR “hospitalization” OR “hospitalised”

Search Concept 3: Risk/predictive factor

(MH “Risk Factors+”) OR “risk factor” OR “cause” OR “causes” OR “predict” OR “predictor”

**Supplementary Text S2: Quality Assessment Criteria**

| **Risk of Bias Item** | **Grading** | **Rationale/Examples** |
| --- | --- | --- |
| **Selection of participants** *(selection bias)* | Low | Sampling frame clearly described with reasonable inclusion/exclusion criteria provided, ideally allowing for inclusion of all participants admitted to the hospital (or specialist service)  Exclusion of those admitted from nursing/residential care and those who did not survive to discharge is considered appropriate |
|  | High | Exclusions based on availability of data (e.g. transfer to other ward/hospital/department; admission to ITU); studies only considering first admission or excluding those with prior admissions/attendances at hospital; exclusion criteria likely to reduce the representativeness of a possible care home population (e.g. comorbidities, end of life care etc.); exclusion of those lost to follow-up; non-consecutive samples if consecutive sample sought |
|  | Unclear | Sampling frame unclear, criteria for inclusion/exclusion not provided or explained |
| **Confounding variables** | Low | Multivariate model accounting for likely possible confounding variables |
|  | High | No consideration of confounding variables; univariate analyses only |
|  | Unclear | Methods for analysis not clearly described or reported |
| **Measurement of exposure** *(performance bias)* | Low | Clearly described method on how data were collected and extracted  Best practice includes description of who performed data extraction, case definitions/descriptions of eligible conditions |
|  | High | Missing data on key predictor variables |
|  | Unclear | Methods for assessing predictor variables not clearly described |
| **Incomplete outcome data**  *(attrition bias)* | Low | Outcomes assessed for all included participants |
|  | High | Missing outcome assessments |
|  | Unclear | Outcome assessment reported as percentages without absolute values being presented, preventing assessment of completeness of outcome reporting |
| **Selective outcome reporting**  *(reporting bias)* | Low | Reporting as per published protocol |
|  | High | Evidence that reporting deviates from publically accessible protocol |
|  | Unclear | No protocol publically available |

Supplementary Table S1: Additional included study population characteristics

| **Study ID/Year** | **Mean Age (SD)** | **Male sex** | **Admission stroke severity** | **Dementia diagnosis** | **Other comorbidities reported** | **Exclusions** |
| --- | --- | --- | --- | --- | --- | --- |
| Béjot 2012 | H 71.6 (16.3)  R 72.7 (15.4)  NH 85.3 (7.5) | 46% | NIHSS Mean (SD)  H 3.8 (3.5)  R 8.6 (5.8)  NH 13.2 (8.5) | 19% (171/913) | Hypertension, diabetes, hypercholesterolemia, atrial fibrillation, smoking, alcohol, TIA, stroke, peripheral arterial disease, heart failure, coronary heart disease & cancer | Nil – population registry of all diagnosed with first or recurrent stroke |
| Brosseau 1996 | 68.9 (14.1) | 50% | NR | NR | NR | Nil – consecutive sample included |
| Ifejika 2015 | C 58.9 (13.8)  SNF 68.2 (12.2) | C 57%  SNF 60% | NIHSS median [IQR]  C 8 [4-13]  SNF 11 [6-15] | NR | NR | Nil – included all those who met admission criteria for neurorehabilitation, irrespective of insurance or ability to pay |
| Kammersgaard 2001 | Inf 77.4 (8.6)  N Inf 73.4 (11.3) | 46% | SSS mean (SD)  Inf 27.4 (15.9)  N Inf 38.3 (16.9) | NR | Diabetes, atrial fibrillation, alcohol, ischaemic heart diease, claudication, hypertension, smoking, previous stroke and previous TIA | Nil – consecutive sample included |
| Koyama 2011 | 69.7 (12) | 61% | FIM mean (SD)  52.55 (22.74) | NR | NR | Excluded those with history of stroke; those who prestroke could not walk unaided and those who were not independent in ADLs |
| Kwan 2007 | Inf 77.9 (10.8)  N Inf 73.2 (12.6) | 49% | SSS median [IQR]  Inf 15 [10-20]  N Inf 20 [18-21] | NR | Atrial fibrillation, hypertension, coronary heart disease, previous cerebrovascular event, diabetes, peripheral vascular disease and smoking | Nil – consecutive sample included |
| Lai 1998 | NH 80.9 (8.6)  RF 70.6 (9.9)  H 69.6 (10.5) | 50% | NR | NR | Hypertension, cardiac arrhythmia, myocardial infarction, diabetes and TIA | Excluded those with a history of stroke and those who lived out of area |
| McManus 2009 | 66.4 (15.9) | 62% | NR | IQCODE >3 28% | History of stroke/TIA, atrial fibrillation, total vascular risk factors, poor vision and poor hearing | Excluded those who had a reduced conscious level, did not speak English and those who were 'delayed' in recruitment |
| Murie-Fernández 2012 | 69 (15) | NR | FIM mean (SD)  81.5 (24.1) | NR | NR | Excluded those who were clinically unstable and not able to sit in a wheelchair for a minimum of 30 minutes |
| Pérez 2016 | 79.1 (7.9) | 49% | NIHSS median [IQR]  9 [4-15] | 21% (79/384) | Smoking status, alcohol consumption, cerebrovascular disease, diabetes, dyslipidaemia and Charlson Index | Excluded those aged <65 years old and those with missing data |

| **Study ID/Year** | **Mean Age (SD)** | **Male sex** | **Admission stroke severity** | **Dementia**  **diagnosis** | **Other comorbidities** | **Exclusions** |
| --- | --- | --- | --- | --- | --- | --- |
| Pinedo 2014 | 71.5 (11.8) | 57% | NR | Mean SPMSQ (SD)  3.0 (2.8) | Charlson Index | Exclusions: admission to rehabilitation >7 weeks after stroke; previous severe disability; lack of cooperation due to cognitive deterioration and/or psychiatric illness; severe systemic illness & lack of informed consent |
| Portelli 2005 | NR | NR | NR | NR | Previous stroke | Nil - Included consecutive admissions to stroke including inpatient stroke |
| Ramirez-Moreno 2008 | NU 87.7 (3.1)  Other 88.9 (3.3) | NU 46%  Other 33% | NR | NU 7%  Other 12% | Hypertension, smoking, hypercholestrolaemia, diabetes, atrial fibrillation, cancer, previous stroke, coronary heart disease, peripheral vascular disease | Nil - Consecutive admissions to both units using hospital registry |
| Rundek 1998 | 69.9 (12.4) | 47% | NR | NR | NR | Excluded recurrent stroke; aged <39; residents out of area; those without a telephone |
| Schlegel 2003 | 65 (15) | 49% | NIHSS: ≤5 56%  6-13 31%  >13 13% | NR | NR | Excluded those with delayed presentation (>24 hours) |
| Treger 2008 | H 69 (12.5)  R 72.4 (11.5)  NH 80.1 (9.2) | 44% | NIHSS score presented as percentages based on discharge disposition | H 6%  R 6%  NH 28% | Hypertension, diabetes, smoking, atrial fibrillation, congestive heart failure, history of myocardial infarction, angina, coronary artery bypass graft, percutaneous coronary intervention, previous stroke, malignancy | Excluded those with missing data |
| Tseng 2015 | D H 65.7 (12.8)  D NH 73.0 (11.7)  V H 66.9 (13.3)  V NH 73.0 (13.0) | 61% | NIHSS: 0-5 59%  6-10 21%  11-15 8%  16-20 5%  >20 7% | NR | Diabetes, snoring, hypertension, heart disease, smoking status and prior stroke | Exclusions included those lost to follow-up along with inpatient deaths and deaths within 3 months grouped together 10.43% of eligible population |
| Turco 2013 | 81.7 (6.4) | 33% | NR | Mean MMSE (SD)  18.1 (7.0) | Malnutrition and depression | Excluded those re-admitted to the acute hospital |

*Notes: ADLs – activities of daily living; C – community; D – derivation; H – home; FIM – Functional Independence Measure; Inf – infection; IQR - interquartile range; MMSE – mini-mental state examination; NH – nursing home; N Inf – no infection; NR – not reported; NU – neurology unit; R – rehab; RF – residential facility; SD – standard deviation; SNF – skilled nursing facility; SPMSQ – short portable mental status questionnaire; SSS – Scandinavian Stroke Scale; TIA – transient ischaemic attack; V – validation. All percentages rounded to nearest whole number*

**Supplementary Figure S1: Risk of Bias Summary Chart**

**
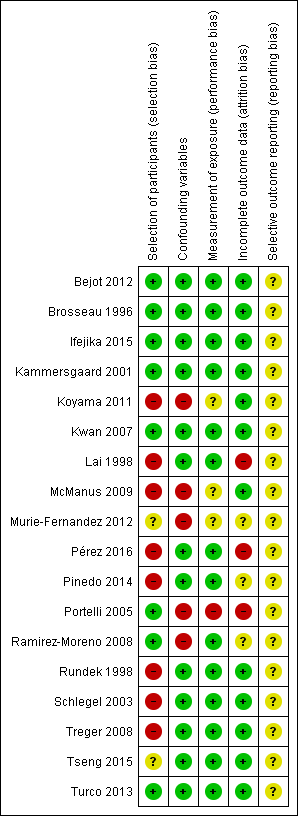
**

**Supplementary Table S2: Complete multivariate predictors of long-term care admission from 11 studies**

| **Predictor** | **Definition** | **Odds Ratio (OR) or Relative Risk (RR)** | **Number in study** | | **Study** |
| --- | --- | --- | --- | --- | --- |
| **AGE** | ≥ 80 at admission *(vs < 60yrs)* | **OR 28.52 (3.47-234.72)** | 1069 | | Béjot |
|  | 60-79 at admission *(vs < 60yrs)* | **OR 9.01 (1.05-77.26)** | 1069 | | Béjot |
|  | ≥ 65 *(vs <65yrs)* | **RR 2.3 (1.1-5.0)** | 573 | | Rundek |
|  | Age ≥75 *(vs 20-44)* | **OR 7.27 (4.27-12.4)** | 21,575 | | Tseng |
|  | Age 65-74 *(vs 20-44)* | **OR 4.74 (2.77-8.10)** | 21,575 | | Tseng |
|  | Age 55-64 *(vs 20-44)* | **OR 2.69 (1.56-4.66)** | 21,575 | | Tseng |
|  | Age 45-54 *(vs 20-44)* | **OR 2.70 (1.54-4.74)** | 21,575 | | Tseng |
|  | Per year | **OR 1.04 (1.01-1.08)** | 346 | | Ifejika |
|  | Per year | **RR 1.2 (1.10-1.20)** | 662 | | Lai |
|  | Per year | OR 1.15 (0.98-1.35) | 94 | | Schlegel |
|  | Per 10 years | **RR 1.93 (1.5-2.5)** | 1583 | | Treger |
|  | | | | | |
| **SEX** | Male | OR 0.93 (0.47-1.82) | 1069 | | Béjot |
|  | Gender *(reference category not stated)* | OR 1.4 (0.7-2.6) | 573 | | Rundek |
|  | Female | RR 1.22 (0.76-1.95) | 1583 | | Treger |
|  | | | | | |
| **LENGTH OF STAY** | >14 days *(vs ≤7)* | OR 1.62 (0.72-3.64) | 1069 | | Béjot |
|  | 7-14 days *(vs ≤7)* | OR 1.07 (0.43-2.66) | 1069 | | Béjot |
|  | Days | OR 0.97 (0.87-1.06) | 346 | | Ifejika |
|  | Days | **RR 1.2 (1.10-1.20)** | 662 | | Lai |
|  | | | | | |
| **STROKE SEVERITY** | NIHSS >20 *(vs ≤5)* | **OR 44.23 (15.79-123.91)** | 1069 | | Béjot |
|  | NIHSS >20 *(vs 0-5)* | **OR 15.9 (12.7-19.9)** | 21,575 | | Tseng |
|  | NIHSS 16-20 *(vs 0-5)* | **OR 10.6 (8.21-13.7)** | 21,575 | | Tseng |
|  | NIHSS >16 *(vs ≤5)* | **RR 38.20 (16.65-87.60)** | 1583 | | Treger |
|  | NIHSS 11-20 *(vs ≤5)* | **OR 9.89 (4.14-23.60)** | 1069 | | Béjot |
|  | NIHSS 11-16 *(vs ≤5)* | **RR 11.45 (6.13-21.40)** | 1583 | | Treger |
|  | NIHSS 11-15 *(vs 0-5)* | **OR 6.64 (5.23-8.43)** | 21,575 | | Tseng |
|  | NIHSS >13 *(vs ≤5)* | **OR 310 (7.8-12434)** | 94 | | Schlegel |
|  | NIHSS 6-13 *(vs ≤5)* | OR 6.4 (0.3-130) | 94 | | Schlegel |
|  | NIHSS 6-10 *(vs ≤5)* | **OR 3.10 (1.44-6.69)** | 1069 | | Béjot |
|  | NIHSS 6-10 *(vs ≤5)* | **RR 2.28 (1.18-4.42)** | 1583 | | Treger |
|  | NIHSS 6-10 (vs 0-5) | **OR 3.00 (2.40-3.75)** | 21,575 | | Tseng |
|  | Total dependence on Barthel *(vs other)* | **OR 2.95 (1.38-6.28)** | 241 | | Pinedo |
|  | Barthel on admission *(0-100)* | **OR 0.94 (0.91-0.97)** | 176 | | Turco |
|  | mRS 4-5 *(at discharge)* | **OR 74.44 (27.35-202.63)** | 1069 | | Béjot |
|  | mRS 2-3 *(at discharge)* | **OR 3.58 (1.46-8.78)** | 1069 | | Béjot |
|  | FIM score | **OR 0.96 (0.94-0.99)** | 152 | | Brosseau |
|  | Admission FIM | OR 0.97 (0.90-1.05) | 346 | | Ifejika |
|  | Discharge FIM | OR 0.96 (0.89-1.04) | 346 | | Ifejika |
|  | | | | | |
| **SOCIAL CIRCUMSTANCES** | Living alone | RR 1.5 (0.9-2.7) | | 573 | Rundek |
|  | Living alone | OR 0.8 (0.2-3.1) | | 94 | Schlegel |
|  | Social support – poor | **OR 2.60 (1.60-4.20)** | | 152 | Brosseau |
|  | Unmarried, divorced or widowed *(vs married)* | **OR 9.17 (1.27-5.77)** | | 241 | Pinedo |
|  | Spouse or siblings *(vs no)* | **OR 0.69 (0.58-0.83)** | | 21,575 | Tseng |
|  | Employed caregiver *(vs no)* | **OR 2.30 (1.92-2.76)** | | 21,575 | Tseng |

| **Predictor** | **Definition** | **Odds Ratio (OR) or Relative Risk (RR)** | **Number in study** | **Study** |
| --- | --- | --- | --- | --- |
| **STROKE SUBTYPE** | Non-lacunar stroke | **RR 2.0 (1.1-3.6)** | 573 | Rundek |
|  | Ischaemic *(vs haemorrhagic)* | OR 0.7 (0.02-20.1) | 94 | Schlegel |
|  | Intracranial haemorrhage (*vs other)* | **RR 3.51 (1.73-7.13)** | 1583 | Treger |
|  | Intracerebral haemorrhage *(vs SVO)* | **OR 2.08 (1.63-2.65)** | 21,575 | Tseng |
|  | Subarachnoid haemorrhage *(vs SVO)* | **OR 2.44 (1.49-4.02)** | 21,575 | Tseng |
|  | Large artery atherosclerosis *(vs SVO)* | **OR 1.45 (1.17-1.81)** | 21,575 | Tseng |
|  | Cardioembolism *(vs SVO)* | **OR 1.67 (1.24-2.25)** | 21,575 | Tseng |
|  | Others *(vs SVO)* | **OR 1.80 (1.41-2.29)** | 21,575 | Tseng |
|  | | | | |
| **STROKE DEFICITS** | Upper and lower limb weakness on admission  *(vs no limb weakness)* | **OR 2.96 (1.36-6.42)** | 1069 | Béjot |
|  | Lower limb weakness on admission *(vs no limb weakness)* | OR 3.15 (0.60-16.56) | 1069 | Béjot |
|  | Deficit in leg | **RR 2.6 (1.20-6.00)** | 662 | Lai |
|  | Upper limb weakness on admission *(vs no limb weakness)* | **OR 3.38 (1.56-7.31)** | 1069 | Béjot |
|  | Deficit in arm | RR 1.5 (0.57-4.00) | 662 | Lai |
|  | Not specified limb weakness on admission  *(vs no limb weakness)* | **OR 3.39 (1.09-10.54)** | 1069 | Béjot |
|  | Ability to walk (yes) | **OR 0.15 (0.031-0.74)** | 152 | Brosseau |
|  | Aphasia on admission  *(vs no limb weakness)* | OR 1.34 (0.72-2.51) | 1069 | Béjot |
|  | Deficit in language | **RR 3.1 (1.40-6.50)** | 662 | Lai |
|  | Deficit in orientation | RR 1.5 (0.66-3.50) | 662 | Lai |
|  | Deficit in facial | **RR 2.5 (1.30-4.80)** | 662 | Lai |
|  | | | | |
| **DEMOGRAPHICS** | Smoking | **OR 0.41 (0.19-0.92)** | 1069 | Béjot |
|  | Smoking | **OR 1.24 (1.07-1.45)** | 21,575 | Tseng |
|  | Race *(Black vs White)* | RR 0.9 (0.4-1.9) | 573 | Rundek |
|  | Race *(Hispanic vs White)* | RR 0.6 (0.3-1.3) | 573 | Rundek |
|  | Education | RR 1.1 (0.6-2.0) | 573 | Rundek |
|  | | | | |
| **COMORBIDITIES & COMPLICATIONS** | Prior stroke | **RR 1.69 (1.05-2.71)** | 1583 | Treger |
|  | Prior stroke | **OR 1.32 (1.14-1.53)** | 21,575 | Tseng |
|  | Heart failure on admission | **OR 2.65 (1.12-6.27)** | 1069 | Béjot |
|  | Cardiac disease | RR 1.2 (0.7-2.1) | 573 | Rundek |
|  | Atrial fibrillation | RR 1.34 (0.76-2.37) | 1583 | Treger |
|  | Heart disease | **OR 0.81 (0.68-0.97)** | 21,575 | Tseng |
|  | Anticoagulant use before admission | **OR 5.55 (1.97-15.66)** | 1069 | Béjot |
|  | Antiplatelet use before admission | **OR 2.28 (1.17-4.44)** | 1069 | Béjot |
|  | Diabetes | **OR 1.26 (1.09-1.47)** | 21,575 | Tseng |
|  | Dementia | **OR 3.21 (1.42-7.24)** | 1069 | Béjot |
|  | Dementia | **RR 4.41 (2.49-7.82)** | 1583 | Treger |
|  | Delirium on admission | **OR 7.23 (4.79-10.91)** | 176 | Turco |
|  | Medical complications | **OR 4.40 (1.10-19.0)** | 152 | Brosseau |
|  | Post-stroke infection | **OR 1.86 (1.00-3.43)** | 439 | Kwan |
|  | CRP on admission | OR 0.96 (0.84-1.10) | 176 | Turco |
|  | High Charlson Index *(vs none or low)* | **OR 2.71 (1.27-5.77)** | 241 | Pinedo |
|  | Malignancy | RR 1.69 (0.86-3.33) | 1583 | Treger |
|  | Snoring (vs not) | **OR 0.81 (0.68-0.98)** | 21,575 | Tseng |
|  | Malnutrition on admission | **OR 5.71 (1.62-20.09)** | 176 | Turco |
|  | Urinary catheter | **OR 4.56 (1.36-15.28)** | 176 | Turco |

*Notes: FIM – Functional Independence Measure; mRS – modified Rankin scale; NIHSS – National Institute for Health Stroke Subscale; SVO – small vessel occlusion*

Text in **bold** denotes statistically significant association. All multivariate data reported in the included studies has been reported.
